# Adult Clinical Perfusion Practice Survey: 2020 results★

**DOI:** 10.1051/ject/2023002

**Published:** 2023-03-24

**Authors:** Breana Lohbusch, Kaylee Olson, Benjamin Magowan, Robert Cherichella, Jeremy Wolverton, Laura Dell’Aiera, Donald S. Likosky, David Fitzgerald

**Affiliations:** 1 Division of Cardiovascular Perfusion, College of Health Professions, The Medical University of South Carolina Charleston SC 29425 USA; 2 Department of Cardiac Surgery, The Center for Healthcare Outcomes & Policy (CHOP), University of Michigan Medical School Ann Arbor MI 48109 USA

**Keywords:** Clinical practice survey, Adult perfusion, Standards, Clinical practice guidelines, Variability

## Abstract

*Background*: Cardiovascular perfusion is a dynamic healthcare profession where new practices are frequently introduced. Despite the emergence of evidence-based clinical practice guidelines, little is known about their dissemination at the institutional level. Clinical practice surveys have been used to identify current trends in perfusion practice in the areas of equipment, techniques, and staffing. This survey aims to describe clinical perfusion practices across adult cardiac surgical programs located in a large, single, geographical region of the United States. *Methods*: Following Institutional Review Board (IRB) approval, an 81-question survey was distributed to 167 adult perfusion programs across the Zone IV region of the American Society of Extracorporeal Technology (AmSECT), a non-profit professional society representing the extracorporeal technology community. Surveys were distributed to chief perfusionists through the Research Electronic Data Capture (REDCap) web-based survey response system. *Results*: Responses were received from 58 of 167 centers across (34.7% response rate). Centrifugal pumps were used at 81% (*n* = 47) of centers and 96.6% (*n* = 56) use an open venous system or hard-shell venous reservoir. Del Nido was the most frequently used cardioplegia strategy with 62.1% (*n* = 36) of centers reporting its use. The use of electronic medical records was reported in 43% (*n* = 25) of centers, while 84.5% (*n* = 49) reported using Cardiopulmonary Bypass (CPB) protocols (>75% of all CPB activities). Extracorporeal Membrane Oxygenation (ECMO) support was reported in 93.1% (*n* = 54) of programs, with 59.2% of programs (*n* = 34) employing a perfusionist as ECMO Coordinator. The *n* + 1 staffing model was reported by 50% (*n* = 29), with 24% supporting the *n* + 1 staffing for after-hours and on-call procedures. *Conclusion*: Clinical practice surveys can be effective tools to inform clinicians about contemporary perfusion practice and identify deviations from professional standards and guidelines. Subsequent surveys may describe trends over time, assess standardization of practice, measure adherence to evidence-based guidelines, and foster improved patient care and outcomes.

## Introduction

Cardiac surgery is one of the most performed inpatient operations in the United States [1]. Despite large-scale improvements in outcomes over the nearly seven decades since the first cardiopulmonary bypass (CPB) procedure [2], appreciable interhospital variability persists in both the conduct of and outcomes associated with CPB [3]. Several efforts have emerged to advance the conduct and safety of CPB, including clinical registries, evidence-based guidelines [4–6] as well as professionally based standards and guidelines [7–9].

Despite the promulgation of these resources for practicing clinicians, little is known about contemporary CPB practices for adult cardiac surgery. While several large-scale clinical registries have emerged, publications to date often focus on a discrete area of practice (e.g., nadir hematocrit during CPB) rather than broadly covering the conduct and practice of CPB. With several topic-specific (e.g., blood management) guidelines have been published, evaluations of the implementation into practice have been infrequent [10–12]. Finally, while The American Society of ExtraCorporeal Technology (AmSECT) has developed professional standards and guidelines for adult CPB, few studies have evaluated real-world practice patterns. This survey aim to describe clinical perfusion practices across adult cardiac surgical programs located in a large, single, geographical region of the United States.

## Materials and methods

Following exempt status approval from the Institutional Review Boards (IRB) of the University of Michigan (HUM00194742) and the Medical University of South Carolina (Pro00107747), an 81-question, the closed-question survey was distributed through the Research Electronic Data Capture (REDCap; Nashville, TN). REDCap is a web-based and secure application for data capture and clinical research database development [15]. The survey topics included program staffing and demographics, equipment, techniques and monitoring, and clinical protocols *(*Appendix). The unit of analysis were center-level adult cardiovascular perfusion programs located in the eastern and mid-Atlantic regions of the United States. A database repository consisting of chief perfusionists (or designee) contact information from programs located in the Zone IV region of AmSECT was previously collected and maintained at the University of Michigan (*Cardiovascular Perfusion Data Repository: Submission ID: REP00000060*). AmSECT Zone IV comprises 15 states and the District of Columbia across the eastern region of the United States, ranging from Maine to South Carolina. A total of 234 cardiac surgical programs were identified in the Zone IV region (Appendix).

The survey questionnaire requested program and procedural data that described clinical practice for the 2020 calendar year. Several questions were also included to identify practice trends over the previous three-year period. Subjects were recruited directly via email invitation on June 8th, 2021. The survey invitation remained open for five weeks, with a closure date of July 16th, 2021. To maximize response rates, non-respondent subjects received up to three notices for participation prior to survey closure. The initial survey was sent to 234 cardiac institutions; however, removing pediatric programs, duplicate or erroneous entries, and missing data resulted in 167 confirmed centers.

Descriptive statistics were used to analyze survey responses. Responses collected from the University of Michigan REDCap repository were de-identified by a database analyst and provided to study investigators. De-identified data were imported into SPSS (IBM SPSS Statistics for Macintosh, Version 27.0, Armonk, NY: IBM Corp.) for analysis and reporting of completed surveys.

## Results

Responses were received from 58 of 167 centers for an overall response rate of 34.7%. Survey responses were received from 13 states with the number of responses between states ranging from 1 to 16 (Table 1). Pennsylvania, New Jersey, New York, and Massachusetts represented 67.3% (*n* = 39) of the responses received.

**Table 1:** Number of respondents and location (*n* = *58*).

State	*n*/*N*	State response rate (%)	Survey response rate (%)
Connecticut	4/11	36.4	6.9
Delaware	2/4	50.0	3.4
Maine	2/3	66.7	3.4
Maryland	3/13	23.1	5.2
Massachusetts	7/12	58.3	12.1
New Hampshire	3/4	75.0	5.2
New Jersey	8/17	47.1	13.8
New York	8/30	26.7	13.8
Pennsylvania	16/55	29.1	27.6
Rhode Island	1/1	100	1.7
South Carolina	2/16	12.5	3.4
Virginia	1/14	7.1	1.7
Vermont	1/1	100	1.7

*n –* number of center respondents, *N* – number of state centers, % – percent responses.

### Staffing and demographics

**Figure 1:**
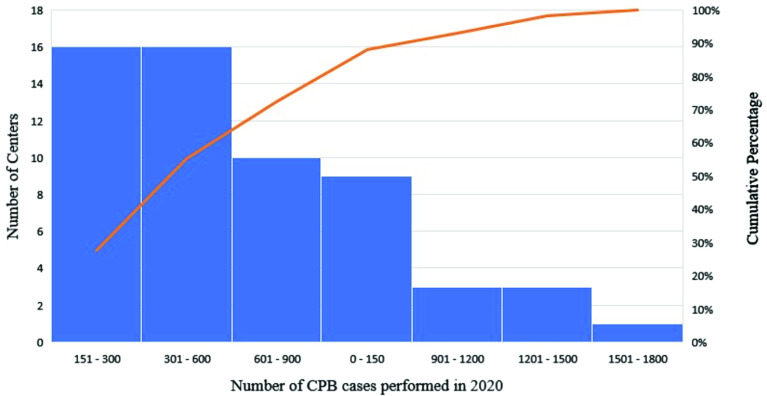
Number and percentage ranges of CPB procedures reported in 2020 (*n* = *58*).

**Figure 2:**
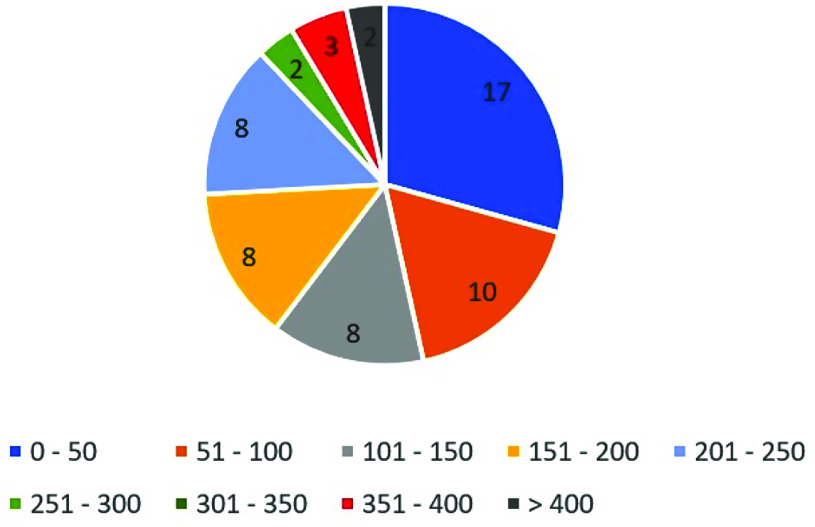
CPB-standby procedures (%) in 2020 (*n* = *58*).

**Figure 3:**
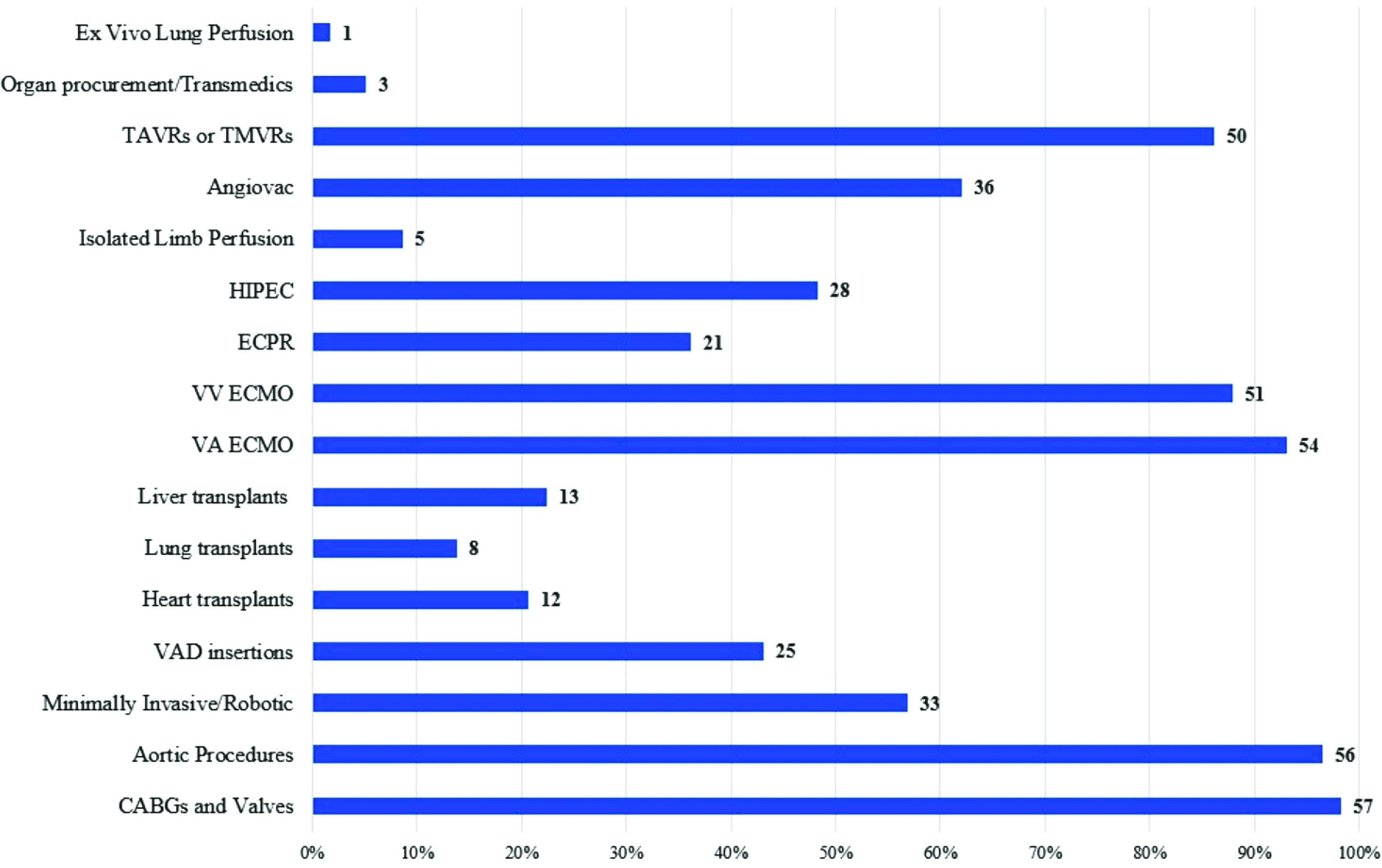
Surgical procedure case type and responding centers that performed them in 2020 (*n* = *58*).

Thirty-two (55.2%) centers reported performing 600 CPB procedures or less in 2020, with 16 (28%) reporting less than 300 (Figure 1). Centers performing 150 CPB standby or less were reported in 35 (60.3%) of responses (Figure 2). The reported distribution of surgical case type is reported in Figure 3. The most frequently reported surgical procedures were coronary artery bypass grafting (CABG) and valve repair/replacement procedures (*n* = 57, 98.3%). Most programs reported the use of both veno-arterial (VA) and veno-venous (VV) extracorporeal membrane oxygenation (ECMO) support (91.4% and 87.9%, respectively). The least frequent surgical procedures reported were ex-vivo lung perfusion (*n* = 1, 1.7%) and organ procurement during transplant (*n* = 3, 5.2%).

**Figure 4:**
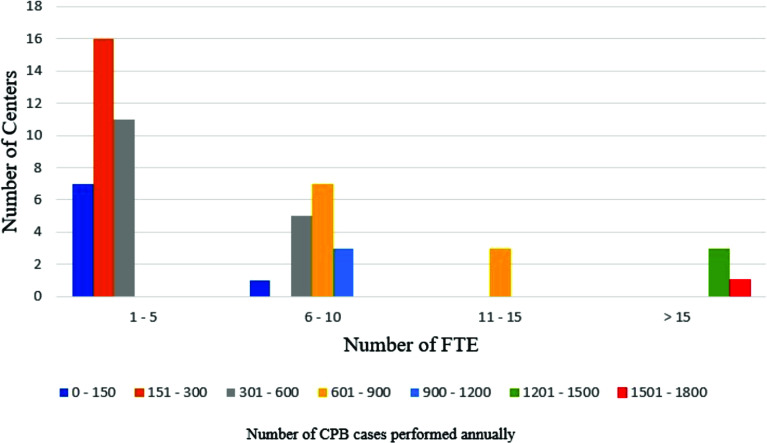
Number of reported Full-Time Equivalents (FTE) perfusionists (*n* = *57*).

The mean number of full-time perfusionists was 6.43, with teams ranging from 1 to 33 perfusionists (Figure 4). Eleven programs (19%) had part-time perfusionists on staff, with 32 (55.2%) reporting the use of per diem or locum tenens coverage over the previous three years. Most respondents (*n* = 43, 72.4%) indicated that the annual number of clinical hours provided by the perfusion team had increased from 2017 to 2020.

**Figure 5:**
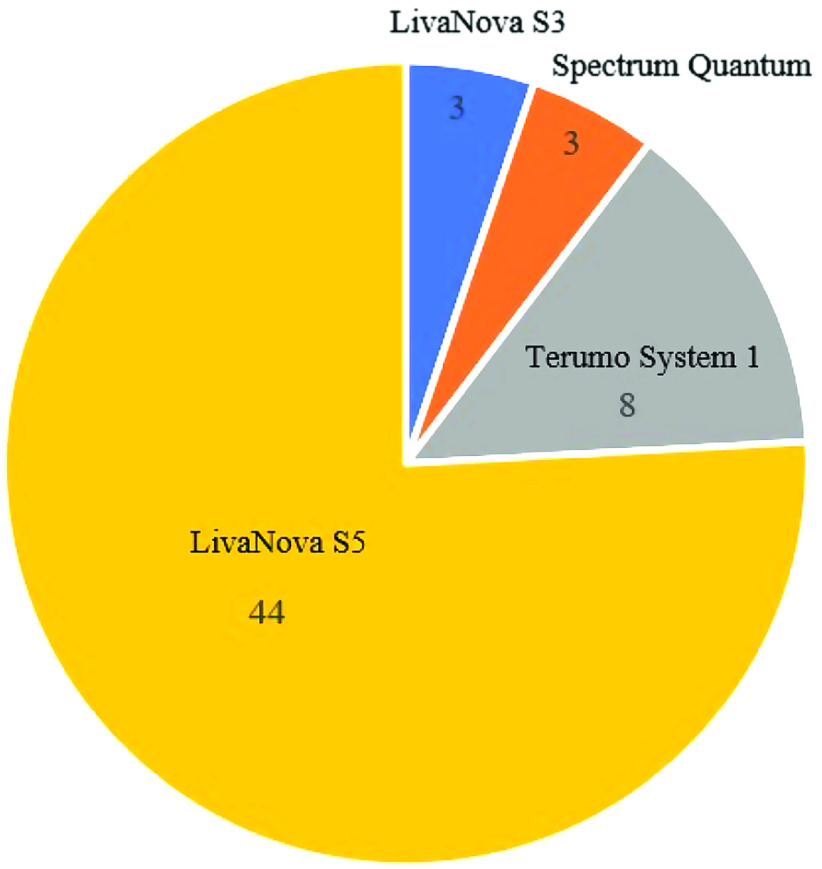
Heart-lung machine type.

The *n* + 1 staffing model, as defined by Guideline 15.1 in the AmSECT Standards and Guidelines for Perfusion Practice, establishes the minimum safe number of perfusion staff required to support operating rooms at any one given time per surgical center [7]. This guideline calls for the presence of one staff member above the number of procedures underway [7]. Respondents reported implementing *N* + 1 during the elective schedule in 50% (*n* = 29) of centers. A total of 4 (6.9%) respondents indicated using more than the *n* + 1, and nine programs (15.5%) reported staffing the minimum 1 perfusionist per procedure. The use of the *n* + 1 model for both elective scheduled and off-hour procedures was reported by 14 (24%) of respondents.

### Equipment

Centrifugal pumps were reported at 47 (81%) centers and 56 (96.6%) utilized an open venous system and hard-shell venous reservoir (Table 2). Most centers reported utilizing arterial and cardioplegia line pressures (100%, *n* = 58), level detectors, and arterial and venous blood temperature monitoring (100% and 98.6%, respectively). Among the lowest reported safety features were one-way valves with centrifugal pumps (*n* = 24, 41.4%), electronic occlusion clamps (*n* = 27, 46.6%), low arterial pump speed alarms (*n* = 29, 50%), and venous reservoir pressure monitoring (*n* = 32, 55.2%). The use of biocompatible coating on all circuitry except cannulas was reported in 47 (81%) responses. Among those using biocompatible circuitry, only 2 (3.8%) were tip-to-tip biocompatible and 3 (5.2%) use bio-coating on limited circuit components.

**Table 2:** CPB circuit components and safety device reporting (*n* = 58).

	Number, (%)
*Arterial pump*	
Roller	10 (17.2)
Centrifugal	47 (81)
Both	1 (1.7)
*Circuit components*	
Open reservoir	56 (96.6)
Closed reservoir	2 (3.4)
*Arterial line filtration*	
Integrated arterial filter/oxygenator	46 (79.3)
External arterial filter only	11 (19)
Both integral and external filters	1 (1.7)
*Safety devices*	
Arterial line pressure monitoring	57 (98.6)
Cardioplegia pressure monitoring	58 (100)
Venous reservoir pressure monitoring	32 (55.2)
Arterial line bubble detector	50 (86.2)
Level detector	56 (96.6)
Arterial blood temperature monitoring	58 (100)
Venous inlet blood temperature monitoring	56 (96.6)
One-way valve in vent lines	55 (94.8)
One-way valve in arterial line (centrifugal only)	24 (41.4)
Servoregulation of arterial pump	34 (58.6)
Electronically activated clamps/occluders	27 (46.6)
Low arterial pump speed alarms	29 (50)
Scavenge line on gas outlet	48 (82.8)
Manual hand cranks in room	56 (96.6)
Back-up gas supply in room	47 (81)
Backup battery supply in room	37 (63.8)
*Perfusion EMR data capture during CPB*	
Yes	26 (44.8)
No	32 (55.2)

Abbreviations: CDPG = cardioplegia; EMR = electronic medical record; CPB: cardiopulmonary bypass.

**Table 3:** CPB filter usage.

Filter type	Always use it (%)	Sometimes use it (%)	Never use it (%)
Pre-Bypass filter	93.1	–	6.9
CPG crystalloid filter	19	–	81
CPG leukocyte filter	1.7	5.2	93.1
Blood transfusion filter	96.6	3.4	–
Systemic leukocyte filter	1.7	12.1	86.2

Abbreviations: CPG = cardioplegia.

The sites for temperature monitoring on patients are listed in Table 4. The most frequently reported core temperature used was a bladder catheter (*n* = 54, 93%). The least reported temperature sources were peripheral temperatures, both skin and tympanic, with an incidence of 1.7% (*n* = 1) and 6.9% (*n* = 4), respectively.

**Table 4:** Patient temperature monitoring sites.

Location of temperature probe	Percent of centers
PA catheter	36.2
Tympanic membrane	6.9
Nasopharyngeal	39.7
Esophageal	29.3
Bladder	93.1
Rectal	12.1
Skin	1.7

Abbreviations: PA = pulmonary artery.

The use of short- and long-term mechanical circulatory support (MCS) devices are reported in Table 5. Respondents were asked to select the devices offered at their centers. Short-term device usage included peripheral access pumps such as the intra-aortic balloon pump (IABP) (100%, *n* = 58), ECMO (93.1%, *n* = 54), and Abiomed Impella (Abiomed, Danvers, MA, USA) (87.9%, *n* = 51). Long-term implantable devices included the Heartmate II and Heartmate III pumps (Abbott Cardiovascular, Plymouth, MN, USA), with a reported usage of 29.3% (*n* = 17) and 39.7% (*n* = 23), respectively.

**Table 5:** Short and durable MCS device utilization. N based on the number of centers that reported performing VAD insertions and ECMO (MCS devices *n* = 58, VAD *n* = 25, ECMO *n* = 54).

	Number (%)
*Short-term MCS*	
Abbott CentriMag	31 (53.4)
Abiomed Impella	51 (87.9)
LivaNova TandemHeart	24 (96)
IABP	58 (100)
ECMO	54 (100)
Other	2 (3.4)
*Durable MCS*	
Abbott HeartMate II™	17 (68)
Abbott HeartMate 3™	23 (92)
HeartWare HVAD	10 (40)
Total Artificial Heart	3 (12)
Berlin Heart	1 (4)

Abbreviations: IABP = intra-aortic balloon pump; ECMO = Extracorporeal membrane oxygenation; MCS = Mechanical circulatory support.

### ECMO utilization and support

**Figure 6:**
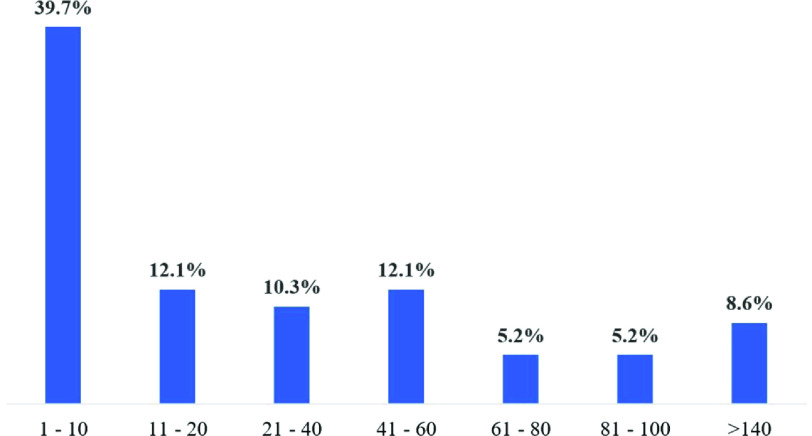
Percent of centers and ECMO procedure volume performed in 2020 (*n* = 54).

Of the 58 centers, 54 (93.1%) offer ECMO at their institution and 59.2% (32/54) had a perfusionist as the ECMO Coordinator. Of those, 39.7% performed 10 ECMO procedures or less (Figure 6). Most centers (87.9%) indicated that the perfusion team was responsible for ECMO initiation and discontinuation, with 91.4% assigned to troubleshooting (Table 6).

**Table 6:** Perfusion team’s clinical responsibilities with ECMO.

ECMO responsibility	Percent of centers
Initiation and Discontinuation	87.9
Troubleshooting	91.4
Direct bedside staffing support	58.6
Rounding model (in house but not bedside)	36.2
Intra-hospital transport	75.9
External transport	69.0

**Table 7:** CPB techniques (*n* = 58).

	Number (%)
*Pulsatile perfusion*	
None	54 (93.1)
During Aortic Cross Clamp Period	3 (5.2)
During Entire CPB	1 (1.7)
*VAVD usage*	
Always	24 (41.4)
Select procedures	29 (50)
Do not use	5 (8.6)
*Carbon dioxide (CO*_*2*_ *) gas priming of the circuit*	
Always	16 (27.6)
Sometimes	4 (6.9)
Never	38 (65.5)
*Prescriptive oxygenation* ^*TM*^	
Yes	18 (31)
No	40 (69)
*Cerebral oximetry monitoring*	
Always	31 (53.4)
Sometimes	22 (37.9)
Never	5 (8.6)
*Blood gas analysis/management*	
Point of care ABG device	30 (51.7)
CPB inline blood gas monitoring	28 (48.3)
Arterial and venous monitoring	18 (31)
*Oxygen delivery monitoring*	
None	31 (53.4)
Manual; calculate during CPB	10 (17.2)
Automated DO2 capture	17 (29.3)
*Blood gas management strategy/mild hypothermia*	
Alpha-stat management	51 (87.9)
pH stat management	6 (10.3)
*Pulsatile perfusion*	
None	54 (93.1)
During aortic cross clamp period	3 (5.2)
During Entire CPB	1 (1.7)
*VAVD usage*	
Always	24 (41.4)
Select procedures	29 (50)
Do not use	5 (8.6)
*Carbon dioxide (CO*_*2*_ *) gas priming of the circuit*	
Always	16 (27.6)
Sometimes	4 (6.9)
Never	38 (65.5)
*Prescriptive oxygenation™*	
Yes	18 (31)
Both	1 (1.7)
*Blood gas management strategy/deep hypothermia*	
Alpha-stat management	33 (56.9)
pH stat management	13 (22.4)
Both	12 (20.7)

Abbreviations: CPB = cardiopulmonary bypass.

### Techniques

Arterial blood gas analysis was performed via point-of-care (POC) handheld device in 51.7% (*n* = 30) of centers. The use of inline blood gas monitoring was reported in 48.3% (*n* = 28). Oxygen delivery (DO2) monitoring during CPB was reported in 46.6% (*n* = 27), with 17 (29.3%) using automated, real-time DO2 monitoring technology and 10 centers (17.2%) performing manual calculations.

During mild to moderate hypothermia cases, 87.9% (*n* = 51) of centers employ alpha-stat for acid-base management, 10.3% (*n* = 6) use pH-stat, and 1.7% (*n* = 1) use a combination of both. In procedures requiring deep to profound hypothermia, 56.9% (*n* = 33) use alpha-stat, 22.4% (*n* = 13) use pH-stat, and 20.7% (*n* = 12) use a combination of both.

Plasmalyte-A was the most used priming solution (Table 8). The most common priming additives were heparin, mannitol, and sodium bicarbonate (Table 8). Other additives reported include dexamethasone, solumedrol, MgSO_4_, or no additives at all (Table 8).

**Table 8:** CPB circuit prime constituents.

	Number (%)
*Base Priming Solution*	
Plasmalyte-A	43 (74)
Lactated Ringers	4 (7)
Normosol-R	9 (16)
Other	2 (3)
*Prime additives*	
Heparin	50 (86)
Sodium Bicarbonate	34 (59)
Albumin 5%	5 (9)
Albumin 25%	22 (38)
Mannitol	41 (71)
Amicar/TXA	18 (31)
Antibiotic	3 (5)
Other	8 (14)

Abbreviations: TXA = Tranexamic acid.

**Figure 7:**
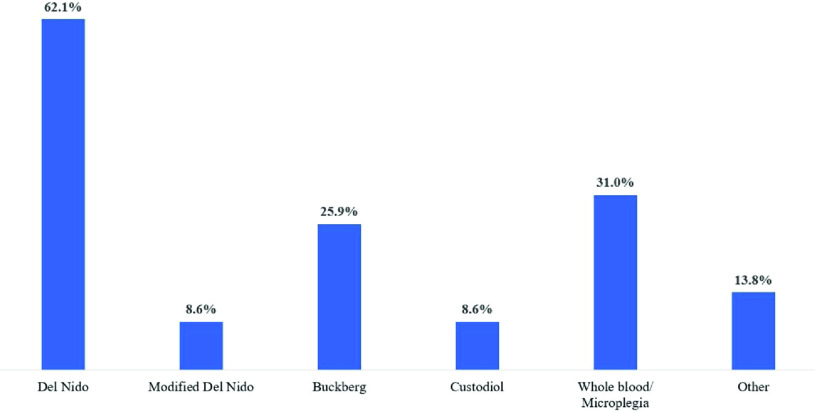
Center-level cardioplegia usage *(n* = 58).

**Figure 8:**
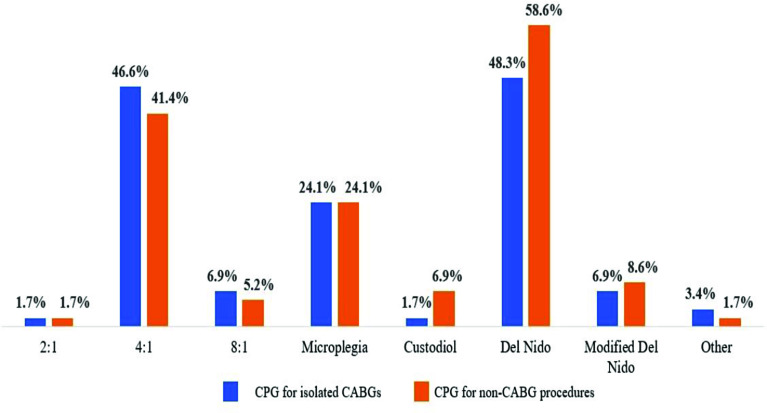
Cardioplegia formulas used for CABG and non-CABG procedures.

del Nido was the most frequently used cardioplegia substrate (62.1%, *n* = 36), followed by whole blood/microplegia (31%, *n* = 18), and Buckberg cardioplegia (25.9%, *n* = 15) (Figures 7 and 8). The type of cardioplegia used varied according to the surgical procedure. Fewer centers used del Nido on isolated CABG procedures (48.3%, *n* = 28). For these procedures, more centers used either 4:1 (46.6%, *n* = 27) or whole blood/microplegia (24.1%, *n* = 12) blood-based solutions. For non-CABG procedures 58.6% (*n* = 34) used del Nido, 41.4% (*n* = 24) used 4:1, and 24.1% (*n* = 14) used microplegia (Figures 7 and 8).

### Blood conservation and anticoagulation strategies

Of the centers that employ autologous circuit priming, 33 (56.9%) use it on most procedures, defined as 75–100% of the time (Table 9). Acute normovolemic hemodilution (ANH) was also reported by 7 (12.1%) centers on select procedures (Table 10). All 58 centers reported using at least one method for salvaging post-CPB circuit blood. Most (93.1%, *n* = 54) transferred post-CPB circuit blood to an autotransfusion device, 55.2% (*n* = 30) reinfused volume into the patient before cannula removal, 24.1% (*n* = 14) used modified ultrafiltration (MUF), 24.1% (*n* = 14) collect pump contents in a bag for reinfusion through a central line, and 1.7% (*n* = 1) use multi-pass ultrafiltration. Anticoagulation management is reported in Table 11. While all centers reported using activated clotting time (ACT) technology, 21.6% used heparin concentration monitoring. Most programs used 480 seconds as a target for CPB support. Protamine dose was calculated using a fixed dose (24.1%, *n* = 14), heparin-protamine titration (27.6%, *n* = 16), and a ratio of heparin given (46.6%, *n* = 27). Pump suction was terminated prior to protamine administration in 41.4% (*n* = 24) of centers, 31% (*n* = 18) terminate suction after 1–25% of protamine has been delivered, and 27.6% (*n* = 15) terminate suction when 26–50% of protamine is administered.

**Table 9:** Autologous prime usage.

Autologous prime is used	Percent of centers
Never	12.1
1–25% of the time	13.8
26–50% of the time	5.2
51–75% of the time	12.1
76–100% of the time	56.9

**Table 10:** ANH usage.

Percent of procedures that use ANH	Percent of centers
Never	43.1
1–25	29.3
26–50	5.2
51–75	10.3
76–100	12.1

**Table 11:** Anticoagulation and hemostasis management.

	Number, (%)
*Minimum ACT For CPB (s)*	
350	4 (6.9)
400	37 (63.8)
450	4 (6.9)
480	10 (17.2)
Other	2 (3.4)
*Target ACT for CPB (s)*	
350	1 (1.7)
400	11 (19)
450	6 (10.3)
480	36 (62.1)
500	1 (1.7)
Other	2 (3.4)
*Anticoagulation Monitoring*	
ACT only	46 (79.3)
ACT/HMS	11 (19)
HMS only	1 (1.7)
*Perioperative Viscoelastic testing*	
Yes	20 (34.5)
No	38 (65.5)
*Protamine Dosing*	
Fixed Dosing	14 (24.1)
HPT Titration	16 (27.6)
Ratio of heparin given	27 (46.6)
Other	1 (1.7)
*CPB Cardiotomy suction termination*	
Start of Protamine	24 (41.4)
1–25% of protamine	18 (31)
26–50%	16 (27.6)

Abbreviations: ACT = activated clotting time; CPB = cardiopulmonary bypass; HMS = Hemostasis Management System; HPT = heparin protamine titration.

### Protocol adoption and adherence

All centers reported using a checklist for some part of their practice (Table 12). Most centers used a checklist for assembly/priming, and initiation of CPB. 65.5% (*n* = 38) of centers keep a dry assembled circuit for routine on-call coverage, and 34.5% (*n* = 20) keep a primed assembled circuit. Once a pump is primed, 4 programs (6.9%) keep it for up to 48 hours, and 10 (17.2%) will use it for up to 72 h. Six centers (10.3%) will keep a primed circuit for up to 1 week. Most centers reported 76–100% of their CPB clinical practice is guided by written departmental protocols (Table 13).

**Table 12:** CPB intraoperative checklist utilization.

Checklist type	Centers that use them (%)
Assembly/priming	96.6
Initiation of CPB	77.6
Weaning/termination of CPB	53.4
Post-CPB	46.6
Transition of care (Hand-off)	36.2
Autotransfusion	43.1
Case Completion	34.5
VAD/MCS	32.8

Abbreviations: CPB = cardiopulmonary bypass; VAD = ventricular assist device; MCS = Mechanical circulatory support.

**Table 13:** Percent of CPB practice supported by institutional protocols.

Percent of practice (%)	Respondents (%)
0–25	1.7
26–50	5.2
51–75	8.6
76–99	44.8
100	39.7

Abbreviations: CPB = cardiopulmonary bypass.

## Discussion

This survey provides an analysis of adult perfusion practice in the Northeast and Mid-Atlantic regions of the United States. We identified substantial variation in multiple areas of perfusion clinical techniques, equipment, staffing, and adoption of clinical practice guidelines. Due to the paucity of comprehensive surveys conducted across US adult clinical perfusion programs, few opportunities have existed to identify and report trends in operative equipment and techniques. The most recent surveys conducted outside of the country may not reflect contemporary US perfusion practice [13, 14]. North American-centric comprehensive surveys date back nearly 27 years [15]. Regional, multi-institutional clinical registries have previously described similar variations in CPB equipment and clinical management strategies; however, the generalizability of these findings is restricted by the number of participating programs [16, 17]. Validated survey tools may broaden the scope of program recruitment and improve our understanding of current perfusion practice. Further, a longitudinal survey design can not only assess clinical trends and guideline adoption, but also identify where the gaps exist for continued consensus development.

The results highlight areas of contrast between published evidence-based guidelines and real-world clinical practice. In 2007, a collaboration between the Society of Thoracic Surgeons (STS) and the Society of Cardiovascular Anesthesiologists (SCA) resulted in a seminal publication of 57 perioperative clinical practice guidelines in cardiac surgical blood conservation and management [18]. Since then, updates to the guidelines were published in 2011 and 2021 [6, 19]. Although several perfusion and intraoperative interventions were assigned high-level recommendations (ACC/AHA Class I and IIa), our findings suggest the application of these techniques have not yet achieved widespread adoption. Examples include the use of autologous circuit priming and perioperative viscoelastic testing. Only 34.5% of respondents reported using perioperative viscoelastic testing, and 69% of centers indicated autologous priming in at least half of all CPB procedures. Both interventions are class I recommendations [16]. Mitigating unwanted variation in clinical practice has been associated with a higher quality of care and lower hospital costs [20, 21]. Specifically, several perfusion-related initiatives have highlighted the importance of evidence-based guidelines adherence, outcome reporting, and the reduction in practice variability [22–24].

Most respondents indicated that the large majority of CPB care plans are supported by institutional protocols. One of the primary responsibilities of a professional society is to develop standards and guidelines of practice to guide the community in safe and effective patient care. The AmSECT Standards and Guidelines, first formed in 1993, aim to define the minimum requirements for safe cardiopulmonary bypass [25]. These guidelines serve as a framework for developing institution-specific CPB protocols [7]. Clinical practice surveys can assist in reporting guideline dissemination and inform key stakeholders of opportunities to support their adoption. For example, Standard 12.1 recommends the discontinuation of CPB cardiotomy suction at the onset of protamine administration to avoid circuit thrombus formation [7]. However, most respondents reported continued suction use after protamine initiation, despite the inability to predict ACT responsiveness. Jansa et al*.* reported a 40% decrease in the ACT value following a partial test dose of protamine, resulting in a value lower than the institutional standard for safe CPB support [26]. While the decision to continue suction use may not ultimately be at the perfusionist’s discretion, reducing these discrepancies in care may require further collaboration and endorsement between surgical and perfusion societies.

The survey results also identify several other areas of non-compliance with professional standards and guidelines. Among them are backup CPB battery availability (64%), backup gas supply (81%), medical gas scavenging of the oxygenator output port (83%), and arterial line bubble detection (86%). Each of these elements is recommended by both AmSECT and the EACTS/EACTA/EBCP guidelines as minimum standards for the safe conduct of CPB. These findings may highlight the importance of understanding the barriers that prevent their adoption. Such barriers may include a lack of awareness of the standards, economic constraints, or perceived benefit of their usage. Professional societies may offer opportunities in facilitating the implementation of these practices at the local level.

Practice surveys may also inform the community about techniques that lack guideline support or clear consensus. Substantial variation is observed across various aspects of cardioplegia use. A recent international survey of cardioplegia practices by Ali and colleagues reported significant variation in myocardial protection strategies [27]. Although blood substrates were the most frequently reported formulas, the dilution ratios and cardioprotective additives were highly variable. Similarly, our survey found both center-level and procedural differences in formulas. In particular, del Nido cardioplegia was the most frequently reported formula in both CABG and non-CABG procedures. The transition to del Nido solution in adults is a recent phenomenon, with the first reported case in 2014 [28]. This was preceded by nearly 25 years of experience in pediatric congenital surgery [29]. The pediatric perfusion survey, first conducted by Groom and colleagues in 1990, has described both domestic and international pediatric perfusion for over 30 years [30–35]. The 2016 survey reported a 74% use of del Nido in North America, a 2.3-fold increase from the 2011 survey results [34, 35]. Similar opportunities for adult surveys performed serially over time would provide valuable insight into the diffusion of new and emerging techniques. Other reported practices lacking clear consensus in the survey included pulsatile perfusion (7%), heparin concentration monitoring during CPB (20.7%), and albumin as a circuit prime additive (47%).

Practice surveys can help describe current and future requirements regarding workforce and staffing. The expansion of adult ECMO services, MCS device implants, and transplant procurement services may necessitate additional perfusion clinical support resources. Respondents reported an increase in clinical workload over the last three years, and most centers indicated the use of part-time and per diem perfusion coverage for relief. Trends in workforce demographics are essential for perfusion supervisors, hospital administrators, and perfusion education programs. Approximately 38% of the certified clinical perfusionists that responded to the 2015–2016 American Board of Cardiovascular Perfusion (ABCP) Perfusion Profile Survey anticipated working 10 more years before retiring [36]. A 2019 survey of perfusion vacancy and turnover estimated rates of 12.3% and 14.7%, respectively [37]. Both rates exceeded those reported in nursing. Considering the timing of these findings and the unknown long-term consequences of the COVID-19 pandemic, workforce survey data is paramount in supporting practitioners and optimizing the quality of care.

There are several limitations to our survey. There are recognized sources of bias in conducting survey research such as sampling bias, compound questioning, recall bias, and respondent misinterpretation of questions. Survey participation was limited to programs located in the Zone IV region of AmSECT, with a response rate of 34.7%. A majority of center responses were received from Northeastern programs. Data collected from these centers may not be generalizable to centers outside of this region. Survey recruitment across additional zones would appreciably increase our understanding of adult perfusion practice. Additionally, the timing of the survey may have been influenced by the Covid-19 pandemic, as centers may have experienced changes in both cardiac surgical and ECMO procedural volume. Recruitment was also restricted to a database containing names and emails of chief perfusionists and managers. The lapse in time between database data collection and survey recruitment may have contributed to lower survey response rates. Many of the non-respondents were attributed to invalid contact information, as several perfusion managers may have left their place of employment prior to the recruitment period. Lastly, this survey did not collect data on all aspects of clinical perfusion service. Examples include ultrafiltration, departmental quality improvement initiatives, or perfusion position vacancy rates.

## Conclusion

Clinical practice surveys can be effective tools in identifying current perfusion staffing, techniques, and equipment utilization. Professional organizations may offer opportunities to promote the recruitment of member center participation. Additional research is warranted to help explain the gaps that may exist between clinical practice guidelines and current perfusion practice. This survey identified several elements of perfusion practice that did not reach thresholds consistent with high-level clinical practice guidelines and professional standards. Longitudinal surveys may describe clinical trends over time and measure adherence to evidence-based and professional standards and guidelines. Benchmarking and trending analysis is necessary to identify areas of improvement, help predict changes in clinical resource management, and foster improved patient outcomes.

## Data Availability

The Informed Consent and Survey Tool used in this study is available in the Appendix of this article.

## References

[1] American Heart Association. Heart procedures and surgeries. Available at: https://www.heart.org/en/health-topics/heart-attack/treatment-of-a-heart-attack/cardiac-procedures-and-surgeries. Accessed March 30, 2022.

[2] Hessel EA 2nd. 2014. A brief history of cardiopulmonary bypass. Semin Cardiothorac Vasc Anesth 18(2), 87–100. 24728884 10.1177/1089253214530045

[3] Likosky DS, Goldberg JB, DiScipio AW, et al. (2012) Variability in surgeons’ perioperative practices may influence the incidence of low-output failure after coronary artery bypass grafting surgery. Circ Cardiovasc Qual Outcomes 5(5), 638–644. 22828825 10.1161/CIRCOUTCOMES.112.967091

[4] Shore-Lesserson L, Baker RA, Ferraris V, et al. (2018) STS/SCA/AmSECT clinical practice guidelines: anticoagulation during cardiopulmonary bypass. J Extra Corpor Technol 50(1), 5–18. 29559750 PMC5850589

[5] Engelman R, Baker RA, Likosky DS, et al. (2015) The Society of Thoracic Surgeons, The Society of Cardiovascular Anesthesiologists, and The American Society of ExtraCorporeal Technology: Clinical practice guidelines for Cardiopulmonary Bypass-Temperature Management during cardiopulmonary bypass. Ann Thorac Surg 100(2), 748–757. 26234862 10.1016/j.athoracsur.2015.03.126

[6] Tibi P, McClure RS, Huang J, et al. (2021) STS/SCA/AmSECT/SABM update to the clinical practice guidelines on patient blood management. J Extra Corpor Technol 53(2), 97–124. 34194077 10.1182/ject-2100053 PMC8220901

[7] American Society of Extracorporeal Technology (AmSECT). Standards and Guidelines for Perfusion Practice. Available at: https://www.amsect.org/p/cm/ld/fid=1617. Accessed March 20, 2022.

[8] DioDato CP, Likosky DS, DeFoe GR, et al. (2008) Cardiopulmonary bypass recommendations in adults: the northern New England experience. J Extra Corpor Technol 40(1), 16–20. 18389661 PMC4680651

[9] Kunst G, Milojevic M, Boer C, et al. (2019) 2019 EACTS/EACTA/EBCP guidelines on cardiopulmonary bypass in adult cardiac surgery. Br J Anaesth 123(6), 713–757. 31585674 10.1016/j.bja.2019.09.012

[10] Likosky DS, FitzGerald DC, Groom RC, et al. (2010) The effect of the perioperative blood transfusion and blood conservation in cardiac surgery Clinical Practice Guidelines of the Society of Thoracic Surgeons and the Society of Cardiovascular Anesthesiologists upon clinical practices. J Extra Corpor Technol 42(2), 114–121. 20648895 PMC4680034

[11] Hessel EA, Groom RC (2021) Guidelines for conduct of cardiopulmonary bypass. J Cardiothorac Vasc Anesth 35(1), 1–17. 32561248 10.1053/j.jvca.2020.04.058

[12] Baker RA, Nikolic A, Onorati F, Alston RP (2020) 2019 EACTS/EACTA/EBCP guidelines on cardiopulmonary bypass in adult cardiac surgery: a tool to better clinical practice. Eur J Cardiothorac Surg 57(2), 207–209. 31942985 10.1093/ejcts/ezz358

[13] Warren OJ, Wallace S, de Wit KL, et al. (2010) Variations in the application of various perfusion technologies in Great Britain and Ireland – a national survey. Artif Organs 34, 200–205. 20447044 10.1111/j.1525-1594.2009.00857.x

[14] Tuble SC, Willcox TW, Baker RA (2009) Australian and New Zealand perfusion survey: management and procedure. J Extra Corp Technol 41, 64–72. PMC4680208 19681302

[15] Silvay G, Ammar T, Reich DL, et al. (1995) Cardiopulmonary bypass for adult patients: a survey of equipment and techniques. J Cardiothorac Vasc Anesth 9, 420–424. 7579112 10.1016/s1053-0770(05)80097-7

[16] Likosky DS, Baker RA, Newland RF, et al. (2018) Is conventional bypass for coronary artery bypass graft surgery a misnomer? J Extra Corpor Technol 50(4), 225–230. 30581229 PMC6296447

[17] Fitzgerald DC, Simpson AN, Baker RA, et al. (2022) Determinants of hospital variability in perioperative red blood cell transfusions during coronary artery bypass graft surgery. J Thorac Cardiovasc Surg 163(3), 1015–1024.e1. 32631660 10.1016/j.jtcvs.2020.04.141 PMC7959104

[18] Ferraris VA, Ferraris SP, et al. (2007) Perioperative blood transfusion and blood conservation in cardiac surgery: the Society of Thoracic Surgeons and The Society of Cardiovascular Anesthesiologists clinical practice guideline. Ann Thorac Surg 83, S27–S86. 17462454 10.1016/j.athoracsur.2007.02.099

[19] Ferraris VA, Brown JR, et al. (2011) 2011 update to the Society of Thoracic Surgeons and the Society of Cardiovascular Anesthesiologists blood conservation clinical practice guidelines. Ann Thorac Surg 91(3), 944–982. 21353044 10.1016/j.athoracsur.2010.11.078

[20] Institute of Medicine (2001). Crossing the Quality Chasm: A New Health System for the 21st Century. Washington, DC: The National Academies Press. 25057539

[21] McGlynn EA, Asch SM, Adams J, et al. (2003) The quality of health care delivered to adults in the United States. N Engl J Med 348, 2635–2645. 12826639 10.1056/NEJMsa022615

[22] Newland RF, Baker RA (2009) Implementing change in perfusion practice: quality improvement vs. experimentation? J Extra Corpor Technol 41(4), 16–20. PMC4813528 20092082

[23] Relle MA, Hutchinson JM, Mattison A, et al. (2014) Predicting adult perfusion practice trends and the adoption of evidence-based practice. J Extra Corpor Technol 46, 53–59. 24779119 PMC4557511

[24] Stammers AH, Trowbridge CC, Pezzuto J, et al. (2009) Perfusion quality improvement and the reduction of clinical variability. J Extra Corpor Technol 41, 48–58. PMC4813536 20092088

[25] Baker RA, Bronson SL, Dickinson TA, et al. (2013) Report from AmSECT’s International Consortium for evidence-based perfusion: American Society of Extracorporeal Technology Standards and Guidelines for Perfusion Practice: 2013. J Extra Corpor Technol 45(3), 156–66. 24303597 PMC4557534

[26] Jansa L, Fischer C, Serrick C, et al. (2022) Protamine test dose: impact on activated clotting time and circuit integrity. Ann Thorac Surg 113(2), 506–510. 33961816 10.1016/j.athoracsur.2021.04.059

[27] Ali JM, Miles LF, Abu-Omar Y, et al. (2018) Global cardioplegia practices: results from the Global Cardiopulmonary Bypass Survey. J Extra Corpor Technol 50(2), 83–93. 29921986 PMC6002645

[28] Mick SL, Robich MP, Houghtaling PL, et al. (2015) del Nido versus Buckberg cardioplegia in adult isolated valve surgery. J Thorac Cardiovasc Surg 149(2), 626–636. 25483897 10.1016/j.jtcvs.2014.10.085

[29] Matte GS, Del Nido PJ (2012) History and use of del Nido cardioplegia solution at Boston Children’s Hospital. J Extra Corpor Technol 44, 98–103. 23198389 PMC4557532

[30] Groom R, Hill A, Akl B, et al. (1990) Pediatric perfusion survey. Proc Am Acad CV Perf 11, 78–84.

[31] Groom RC, Hill AG, Kurusz M, et al. (1995) Paediatric perfusion practice in North America: an update. Perfusion 10(6), 393–401. 8747896 10.1177/026765919501000603

[32] Cecere G, Groom R, Forest R (2002) A 10-year review of pediatric perfusion practice in North America. Perfusion 17, 83–89. 11958308 10.1191/0267659102pf542oa

[33] Groom R, Froebe S, Martin J, et al. (2005) Update on pediatric perfusion practice in North America: 2005 survey. J Extra Corpor Technol 37, 343–350. 16524149 PMC4680823

[34] Harvey B, Shann KG, Fitzgerald D, et al. (2012) International pediatric perfusion practice: 2011 survey results. J Extra Corpor Technol 44(4), 186–93. 23441558 PMC4557559

[35] Walcƶak A, Klein T, Voss J, et al. (2021) International pediatric perfusion practice: 2016 survey results. J Extra Corpor Technol 53(1), 7–26. 33814602 10.1182/ject-2000033 PMC7995632

[36] Turnage C, DeLaney E, Kulat B, et al. (2017) A 2015–2016 survey of American board of cardiovascular perfusion certified clinical perfusionists: perfusion profile and clinical trends. J Extra Corpor Technol 49(3), 137–149. 28979037 PMC5621577

[37] Colligan M (2020) Results of the 2019 survey on perceptions of vacancy and turnover among perfusionists in the United States. J Extra Corpor Technol 52(1), 27–42. 32280142 10.1182/JECT-2000001 PMC7138123

